# TRAUMA THROUGH THE LIFE CYCLE IN AN IMMIGRANT POPULATION

**DOI:** 10.1093/geroni/igz038.3409

**Published:** 2019-11-08

**Authors:** Gabriella Dong, Stephanie M Bergren

**Affiliations:** Rutgers, The State University of New Jersey, New Brunswick, New Jersey, United States

## Abstract

The majority of studies on traumatic events have focused on either children or younger adults, while traumatic events in older adults have not been sufficiently investigated. Older immigrants encountered a wide range of traumatic events across the life span, before and after immigration, in the origin and host countries. This study aims to provide a descriptive epidemiology of lifetime traumatic events in older Chinese Americans. The data were drawn from the Population Study of Chinese Elderly in Chicago (PINE) in 2017-2019, with a sample size of 3,126. Traumatic events were evaluated by natural disasters, personal and historical events. After examining the lifetime prevalence of natural disasters, we found typhoon (64.46%) has the highest prevalence, followed by earthquake (39.81%) and tornado (7.25%). In terms of personal events, death of a loved one (69.78%) was the most prevalent, followed by robbery (12.57%), physical assault (5.36%), fire (5.29%), divorce (5.16%), cancer (5.10%), falsely accused (2.15%), homeless (1.57%), sexual assault (0.99%), and imprisonment (0.74%). In addition, 18.91% of women experienced abortion and 11.25% of women experienced miscarriage,. With respect to historical events, most participants experienced the Cultural Revolution (73.27%), the Great Leap Forward (62.71%), and famine (60.01%). A small proportion experienced the Japanese invasion of China (27.14%), Tiananmen Square protests (7.86%), and the Vietnam war (4.78%). In our sample, women were more likely than men to encounter traumatic life events. Further studies could examine the influence of cumulative exposure to natural disasters, personal events and historical events on health outcomes of older immigrants.

